# Predictors of In-Hospital Death in Patients With Acute Myocardial Infarction

**DOI:** 10.7759/cureus.43392

**Published:** 2023-08-12

**Authors:** Osamu Sasaki, Toshihiko Nishioka, Yoshiro Inoue, Ami Isshiki, Hideki Sasaki

**Affiliations:** 1 Cardiology, Saitama Medical Center, Saitama Medical University, Kawagoe, JPN; 2 Internal Medicine, Mombetsu General Hospital, Mombetsu, JPN; 3 Cardiovascular Surgery, Nagoya City University East Medical Center, Nagoya, JPN

**Keywords:** pre and post percutaneous coronary intervention, in-hospital death, multi-vessel disease, iabp/pcps, left main trunk lesion, acute myocardial infarction

## Abstract

Objective: Factors such as age, vital signs, renal function, Killip class, cardiac arrest, elevated cardiac biomarker levels, and ST deviation predict survival in patients with acute myocardial infarction (AMI). However, the existing risk assessment tools lack comprehensive consideration of catheter-related factors, and short-term prognostic predictors are unknown. This study aimed to clarify in-hospital prognostic predictors in hospitalized patients with AMI.

Methods: Five hundred and thirty-six patients who underwent percutaneous coronary intervention (PCI) for AMI were divided into non-survivor (n = 36) and survivor (n = 500) groups. Coronary risk factors, laboratory findings, angiographic findings, and clinical courses were compared between the two groups. Multiple logistic regression was used to analyze in-hospital death in pre- and post-PCI phases.

Results: In the pre-PCI phase, multiple logistic regression analysis revealed several predictors of in-hospital death, including systolic blood pressure [odds ratio (OR) = 0.985, p = 0.023)], Killip class ≥2 (OR = 14.051, p <0.001), and chronic kidney disease (OR = 4.859, p = 0.040). In the post-PCI phase, multiple logistic regression analysis revealed additional predictors of in-hospital death, including Killip class ≥2 (OR = 5.982, p = 0.039), presence of lesions in the left main trunk (OR = 51.381, p = 0.044), utilization of intra-aortic balloon pumps and percutaneous cardiopulmonary support (OR = 6.141, p = 0.016), and presence of multi-vessel disease (OR = 6.323, p = 0.022).

Conclusion: Predictors of in-hospital death in AMI extend beyond conventional risk factors to include culprit lesions, mechanical support, and multi-vessel disease that manifest post-PCI.

## Introduction

Acute myocardial infarction (AMI) is a life-threatening condition, and our clinical practice has led to the emergence of several useful risk assessment tools for evaluating in-hospital prognosis. These include the TIMI (Thrombolysis in Myocardial Infarction), GRACE (Global Registry of Acute Cardiac Events), and CADILLAC (Controlled Abciximab and Device Investigation to Lower Late Angioplasty Complications) risk scores [1-3], which offer convenient methods for assessing the risk of acute coronary syndrome. These tools incorporate various evaluation parameters, such as age, coronary risk factors, prior history of coronary artery disease, aspirin use, suspected angina pectoris symptoms, ST deviation, elevated cardiac biomarkers, heart rate, systolic blood pressure, serum creatinine levels, Killip class, cardiac arrest, age ≥65 years, baseline left ventricular ejection fraction ≤40%, anemia, renal insufficiency, triple-vessel disease, and post-procedural TIMI flow grade. However, it is worth noting that these risk scores do not comprehensively encompass factors directly related to percutaneous coronary intervention (PCI). Given these considerations, the primary objective of our study was to elucidate the predictive factors associated with in-hospital mortality, with a specific focus on the variables that arise after PCI.

## Materials and methods

Study population

This study included 536 patients with AMI who underwent primary PCI at our institution between April 2010 and July 2016. Patients who had previously undergone coronary artery bypass grafting (CABG), and those who experienced cardiac arrest prior to hospitalization but did not undergo PCI were excluded. The enrolled patients were subsequently divided into two groups: the survivor (S) group, consisting of patients who were discharged from the hospital, and the non-survivor (NS) group, consisting of patients who died during their hospital stay. The Institutional Review Board of Saitama Medical Center, Saitama Medical University approved the study (approval number 1551), and all patients provided written informed consent to participate.

Definitions

AMI was defined according to the European Society of Cardiology (ESC)/American College of Cardiology Foundation (ACCF)/American Heart Association (AHA)/World Health Federation (WHF) Task Force for the Universal Definition of Myocardial Infarction [4]. Dyslipidemia was defined as a low-density lipoprotein cholesterol (LDL-C) level >140 mg/dL, high-density lipoprotein cholesterol (HDL-C) level <40 mg/dL, triglyceride level >150 mg/dL, or use of cholesterol-lowering medication. Smoking was defined as current smoking or smoking cessation less than one month before enrollment. Hypertension was defined as systolic blood pressure (SBP) >140 mmHg and/or diastolic blood pressure >90 mmHg or use of antihypertensive drugs. Patients with diabetes mellitus had a confirmed diagnosis or were taking insulin or oral hypoglycemic agents at the time of enrollment. Obesity was defined as body mass index (BMI) >25 kg/m2. BMI was calculated as weight (kg) divided by height squared (m2). Chronic kidney disease (CKD) was defined as a reduced estimated glomerular filtration rate (eGFR) <60 mL/min per 1.73 m2. Multi-vessel disease was defined as the presence of >75% stenosis in at least two significant coronary arteries.

Evaluation of electrocardiogram

Upon admission to the emergency department, 12-lead electrocardiogram (ECG) data were obtained as soon as possible, and the following parameters were compared between the groups: heart rate, QRS width, and ST-segment elevation. QRS duration was measured using a caliper in the lead with the longest QRS duration. ST-segment elevation was measured 60 ms after the J point, and a significant ST-segment change was defined as deviation of >0.05 mV from the baseline.

Angiographic evaluation and PCI procedure

After thrombectomy of the culprit lesion, catheter interventions such as standard balloon angioplasty and primary stenting were performed. An initial bolus of unfractionated heparin 3,000 units was administered, followed by 5,000 units immediately before PCI, with continuous infusion for three days. Dual antiplatelet therapy with aspirin, ticlopidine, or clopidogrel was continued in all patients. Initiation of mechanical support, such as intra-aortic balloon pumping (IABP) and percutaneous cardiopulmonary support (PCPS), was determined at the discretion of the attending cardiologist. All angiograms were evaluated by consensus of two independent interventional cardiologists, who were blinded to the study protocol and patient background.

Hospital course and follow-up

We assessed in-hospital clinical events, including elevated creatine kinase (CK) levels, in-hospital death, and the occurrence of CABG during the hospitalization period. Furthermore, we performed a comparative analysis to evaluate the frequency of IABP and PCPS among the different groups.

Statistical analysis

All data were analyzed using the SPSS software (version 22.0; IBM Corp., Armonk, NY, USA). Continuous variables are expressed as means ± SD and categorical variables as numbers and ratios (%). Continuous variables were compared between the NS and S groups using an unpaired t-test, and categorical variables were assessed using the chi-square test. P <0.05 was considered statistically significant. Multiple logistic regression analyses were performed to analyze in-hospital deaths in this population in the pre- and post-PCI phases. Odds ratios (OR) and 95% confidence intervals (CI) for in-hospital death were calculated from the outcomes of multiple logistic regression models after adjustment for potential confounders of age, sex, hypertension, dyslipidemia, diabetes mellitus, CKD, smoking status, hyperuricemia, SBP, heart rate, ST elevation in anterior leads, QRS width, Killip class ≥2, culprit lesion, mechanical support (such as IABP and PCPS), and peak CK levels.

## Results

Baseline characteristics of study patients

Table 1 presents the baseline patient characteristics. The mean age was 66.8 ± 12.0 years, and 412 (76.9%) patients were males. The frequencies of sex, hypertension, dyslipidemia, diabetes mellitus, obesity, smoking, medications such as angiotensin-converting enzyme inhibitor/angiotensin II receptor blockers, calcium channel blockers, plasma hemoglobin A1C (HbA1C) (%), and BMI did not differ significantly between the two groups. However, the frequencies of hyperuricemia, CKD, Killip class ≥2, ST elevation in anterior leads were significantly higher in the NS group than in the S group (41.7% vs 12.4%, p <0.001; 100% vs 37.8%, p <0.001; 88.9% vs 15.0%, p <0.001; 55.6% vs 30.4%, p = 0.001, respectively). The heart rate and QRS width were significantly higher in the NS group than in the S group (94.4 ± 27.9 bpm vs 74.2 ± 18.6 bpm, p <0.001; 137.1 ± 34.3 ms vs 110.5 ± 22.8 ms p <0.001, respectively). The SBP, eGFR, and plasma LDL-C and HDL-C levels were significantly lower in the NS than in the S group (79.1 ± 46.6 mmHg vs 142.2 ± 35.5 mmHg, p <0.001; 39.7 ± 18.0 mL/min/1.73 m2 vs 66.1 ± 33.4 mL/min/1.73 m2, p <0.001; 98.5 ± 43.9 mg/dL vs 114.1 ± 36.2 mg/dL, p = 0.033; 34.0 ± 12.9 mg/dL vs 43.6 ± 11.5 mg/dL, p <0.001, respectively). 

**Table 1:** Patients’ characteristics. ACE-I, angiotensin-converting enzyme inhibitor; ARB, angiotensin Ⅱ receptor blocker; BMI, body mass index; CCB; calcium channel blocker; CKD, chronic kidney disease; eGFR, estimated glomerular filtration rate; HDL-C, high density lipoprotein cholesterol; LDL-C, low density lipoprotein cholesterol; SBP, systolic blood pressure; STE, ST elevation.

	All patients (n = 536)	Non-survivors (n = 36)	Survivors (n = 500)	p-value
Age (years)	66.8 ± 12.0	69.0 ± 11.2	66.7 ± 12.0	0.269
Male gender	412 (76.9%)	27 (75.0%)	385 (77.0%)	0.783
Hypertension	394 (73.5%)	24 (66.7%)	370 (74.0%)	0.355
Dyslipidemia	351 (65.5%)	22 (61.1%)	329 (65.8%)	0.567
Diabetes Mellitus	199 (37.1%)	15 (41.7%)	184 (36.8%)	0.559
Obesity (BMI>25)	192 (35.8%)	8 (22.2%)	184 (36.8%)	0.078
Smoking	210 (39.2%)	10 (27.8%)	200 (40.0%)	0.146
Hyperuricemia	77 (14.4%)	15 (41.7%)	62 (12.4%)	<0.001
CKD	222 (41.4%)	36 (100%)	189 (37.8%)	<0.001
SBP (mmHg)	137.8 ± 39.7	79.1 ± 46.6	142.2 ± 35.5	<0.001
Killip class ≥2	107 (19.9%)	32(88.9%)	75 (15.0%)	<0.001
Heart Rate (bpm)	75.6 ± 20.0	94.4 ± 27.9	74.2 ± 18.6	<0.001
QRS width (ms)	112.3 ± 24.7	137.1 ± 34.3	110.5 ± 22.8	<0.001
STE in anterior leads	172 (32.1%)	20 (55.6%)	152 (30.4%)	0.001
Medications				
Statin	119 (22.2%)	5 (13.9%)	116 (23.2%)	0.196
ACE-I/ARB	152 (28.4%)	6 (16.7%)	146 (29.2%)	0.107
β-blocker	82 (15.3%)	3 (8.3%)	79 (15.8%)	0.229
CCB	168 (31.3%)	8 (22.2%)	160 (32.0%)	0.221
eGFR (ml/min/1.73m^2^)	64.3 ± 33.2	39.7 ± 18.0	66.1 ± 33.4	<0.001
LDL-C (mg/dL)	113.3 ± 36.7	98.5 ± 43.9	114.1 ± 36.2	0.033
HDL-C (mg/dL)	43.1 ± 11.7	34.0 ± 12.9	43.6 ± 11.5	<0.001
HbA1c (%)	6.4 ± 2.8	6.8 ± 1.8	6.4 ± 2.8	0.484
BMI (kg/m^2^)	24.1 ± 3.5	24.2 ± 3.4	22.9 ± 4.6	0.060

Angiographic findings and clinical course

Table 2 shows the angiographic findings and clinical course. The frequencies of left main coronary artery disease as culprit lesions, multi-vessel disease, IABP, and PCPS requirements were significantly higher in the NS than in the S group (22.2% vs 0.4%, p <0.001; 61.1% vs 35.0%, p = 0.001; 63.9% vs 6.0%, p <0.001, respectively). The peak CK values were significantly higher in the NS group than in the S group (5582.8 ± 5841.4 U/L vs 1928.7 ± 2144.8 U/L, p <0.001). The frequency of CABG during hospitalization did not differ between the two groups.

**Table 2:** Angiographic findings and clinical course. CABG, coronary artery bypass grafting; CK, creatine kinase; IABP, intraaortic balloon pumping; LAD, left anterior descending; LCx, left circumflex; LMT, left main trunk; PCPS, percutaneous cardiopulmonary support; RCA, right coronary artery.

	All patients (n = 536)	Non-survivors (n = 36)	Survivors (n = 500)	p-value
Culprit artery				
LMT	10 (1.9%)	8 (22.2%)	2 (0.4%)	<0.001
LAD	236 (44.0%)	13 (36.1%)	223 (44.6%)	0.32
LCx	87 (16.2%)	4 (11.1%)	83 (16.6%)	0.375
RCA	180 (33.6%)	10 (27.7%)	170 (34.0%)	0.445
Others	23 (4.3%)	1 (2.7%)	22 (4.4%)	0.642
Multi vessel disease	196 (36.6%)	22 (61.1%)	175 (35.0%)	0.001
IABP and or PCPS	53 (9.9%)	23 (63.9%)	30 (6.0%)	<0.001
Peak CK (U/L)	2175.1 ± 2715.0	5582.8 ± 5841.4	1928.7 ± 2144.8	<0.001
CABG during hospitalization	19 (3.5%)	1 (2.7%)	18 (3.6%)	0.796

Predictors of in-hospital death

Table 3 shows multiple regression analysis of in-hospital deaths in patients with AMI. In the pre-PCI phase, multiple logistic regression analysis revealed that SBP (OR = 0.985, p = 0.023), Killip class ≥2 (OR = 14.051, p <0.001), and CKD (OR = 4.859, p = 0.040) emerged as significant independent predictors for in-hospital mortality. Conversely, in the post-PCI phase, multiple logistic regression analysis demonstrated that Killip class ≥2 (OR = 5.982, p = 0.039), left main trunk (LMT) involvement (OR = 51.381, p = 0.044), utilization of IABP and PCPS (OR = 6.141, p = 0.016), and presence of multi-vessel disease (OR = 6.323, p = 0.022) independently predicted in-hospital mortality. The area under the receiver operating characteristic curve (AUC), which evaluates the goodness-of-fit in a regression analysis, demonstrated high pre- and post-PCI values of 0.951 and 0.969, respectively.

**Table 3:** Multiple logistic regression analysis of in-hospital death (pre- and post-PCI). Adjusted for age, sex, ST elevation in anterior leads, smoking, hyperuricemia, hypertension, dyslipidemia, diabetes mellitus, and obesity. AUC, area under the curve; CI, confidence interval; CK, creatine kinase; CKD, chronic kidney disease; IABP, intraaortic balloon pumping; LMT, left main trunk; OR, odds ratio; PCI, percutaneous coronary intervention; PCPS, percutaneous cardiopulmonary support; SBP, systolic blood pressure

	Pre-PCI			Post-PCI		
	OR	95% CI	p-value	OR	95% CI	p-value
SBP	0.985	0.972-0.998	0.023	0.987	0.970-1.005	0.173
Killip class ≥2	14.051	3.364-58.58	<0.001	5.982	1.086-32.92	0.039
QRS width	1.016	0.998-1.034	0.071	1.021	0.997-1.045	0.078
Heart Rate	1.019	0.997-1.042	0.083	1.021	0.989-1.055	0.190
CKD	4.859	1.071-22.03	0.040	5.505	0.876-34.57	0.068
LMT				51.381	1.092-2415	0.044
IABP/PCPS				6.141	1.386-27.20	0.016
Multi-vessel disease				6.323	1.304-30.66	0.022
Peak CK				1.000	0.999-1.000	0.438
AUC	0.951			0.969		

## Discussion

In this study, we identified two important clinical issues. First, consistent with the findings of previous studies, the pre-PCI predictors of in-hospital mortality included systolic blood pressure, Killip class ≥2, and renal function. However, the post-PCI predictors of in-hospital mortality in this study included lesion characteristics such as presence of LMT lesions and multi-vessel disease, as well as severity indices such as Killip class ≥2 and utilization of IABP or PCPS. Second, although no notable disparities were observed in the distribution of coronary artery risk factors between the two groups, it is important to highlight the substantial prevalence of these risk factors. Hypertension was observed in 73.5% of the population, dyslipidemia in 65.5%, diabetes mellitus in 37.1%, and smoking in 39.2%.

Predictors of in-hospital death

In this study, incorporation of post-PCI variables resulted in notable alterations in multiple regression outcomes. Presence of LMT lesions, multi-vessel disease, mechanical support (utilization of IABP or PCPS), and severity of AMI (Killip class ≥2), were extracted as important factors for in-hospital death. The significantly high mortality rate of 80% observed in our study population, specifically among patients with LMT lesions, is particularly noteworthy. This finding underscores an exceedingly unfavorable prognosis, which aligns with the findings of previous reports on this subject [5-9]. Resolving this predicament is challenging, given that LMT obstruction leads to widespread myocardial ischemia, necrosis within the cardiac tissue, and consequential catastrophic damage.

Utilization of IABP or PCPS emerged as a predictor of in-hospital mortality in this study. Although a meta-analysis of previous cohort studies has reported reduction in mortality in patients with cardiogenic shock with utilization of IABP [10], in the IABP-SHOCKⅡ trial, which is a randomized study involving patients with ST elevated myocardial infarction, utilization of IABP did not demonstrate any discernible enhancement in the 30-day mortality rate [11]. However, in the realm of practical clinical practice, healthcare professionals face the challenging task of making critical decisions regarding the utilization of IABP in patients presenting with compromised hemodynamics. This outcome highlights the real and intricate nature inherent in clinical settings pertaining to PCI. Furthermore, the observation that Killip class and presence of multi-vessel disease emerged as significant predictors of in-hospital death, is consistent with findings reported previously [2,3].

While our study encompassing post-PCI variables did not identify renal function and QRS width on ECG as significant predictors, it is important to acknowledge that these factors were clinically significant. Previous reports have demonstrated the utility of renal function [2,3] and QRS width [12] as predictive markers of in-hospital mortality in patients with AMI. However, the OR for the QRS width did not yield a substantial magnitude in our study.

Our findings reveal that the predictors of in-hospital mortality in AMI extend beyond conventional risk factors, to include factors that manifest in the post-PCI phase. Specifically, presence of lesions in the LMT and utilization of IABP or PCPS, in conjunction with the coexistence of multi-vessel disease, may independently contribute to an elevated risk of mortality.

Coronary risk factors

Traditional coronary risk factors, including hypertension, dyslipidemia, diabetes mellitus, and smoking, have not emerged as significant risk factors for in-hospital mortality. This observation could be attributed to the fact that the study population comprised individuals who had already developed AMI, potentially influencing the association between these risk factors and outcomes. In our population, the prevalence of hypertension and dyslipidemia was >60%, the prevalence of diabetes mellitus was >30%, and the prevalence of therapeutic intervention was comparatively modest, with statin utilization at 22.2%, and the administration of antihypertensive agents ranged between 15.3% and 31.3%. The Takashima AMI registry [13], an esteemed Japanese cohort study, revealed a progressive increase in the age-adjusted incidence of AMI from the 1990s to the 2000s. And, the Hisayama study [14] showed a decline in the incidence of stroke, which was attributed to the successful management of blood pressure. However, in contrast to the notable change in the incidence of stroke, the incidence of AMI did not exhibit a significant alteration. This study highlights poor control of metabolic factors such as glucose intolerance, dyslipidemia, and obesity as potential contributors to this trend. However, previous reports have demonstrated the pivotal role of effective management of hypertension, dyslipidemia, and diabetes mellitus in reducing cardiovascular events, thus emphasizing the importance of this task [15-20]. Considering both previous findings and our findings, there is a pressing need to emphasize and reinforce the management of coronary risk factors.

Limitations

Our study has some limitations. First, it was a single-center, cross-sectional, observational investigation, which differed significantly from a randomized controlled trial. Second, our findings cannot be extrapolated to patients who previously underwent CABG, experienced cardiac arrest, or did not undergo PCI. Third, we did not have information on echocardiographic parameters, and post-procedural TIMI flow grade was not adequately evaluated or recorded during the acute phase.

## Conclusions

Pre-PCI, systolic blood pressure, Killip class ≥2, and CKD were predictors of in-hospital death. Post-PCI, Killip class ≥2, LMT lesions, utilization of IABP/PCPS, and multi-vessel disease were predictors of in-hospital death. This study highlights the necessity for reevaluation of risk assessment pertaining to in-hospital death following AMI, both in the pre- and post-PCI phases.

## References

[1] Antman EM, Cohen M, Bernink PJ (2000). The TIMI risk score for unstable angina/non-ST elevation MI: a method for prognostication and therapeutic decision making. JAMA.

[2] Granger CB, Goldberg RJ, Dabbous O (2003). Predictors of hospital mortality in the global registry of acute coronary events. Arch Intern Med.

[3] Halkin A, Singh M, Nikolsky E (2005). Prediction of mortality after primary percutaneous coronary intervention for acute myocardial infarction: the CADILLAC risk score. J Am Coll Cardiol.

[4] Thygesen K, Alpert JS, Jaffe AS (2012). Third universal definition of myocardial infarction. Circulation.

[5] Yip HK, Wu CJ, Chen MC, Chang HW, Hsieh KY, Hang CL, Fu M (2001). Effect of primary angioplasty on total or subtotal left main occlusion: analysis of incidence, clinical features, outcomes, and prognostic determinants. Chest.

[6] Luca GD, Suryapranata H, Thomas K (2003). Outcome in patients treated with primary angioplasty for acute myocardial infarction due to left main coronary artery occlusion. Am J Cardiol.

[7] Lee SW, Hong MK, Lee CW (2004). Early and late clinical outcomes after primary stenting of the unprotected left main coronary artery stenosis in the setting of acute myocardial infarction. Int J Cardiol.

[8] Izumikawa T, Sakamoto S, Takeshita S, Takahashi A, Saito S (2012). Outcomes of primary percutaneous coronary intervention for acute myocardial infarction with unprotected left main coronary artery occlusion. Catheter Cardiovasc Interv.

[9] Sasaki O, Sasaki H (2023). Electrocardiographic QRS findings upon admission can predict prognosis of acute myocardial infarction caused by occlusion of left main coronary artery. Cureus.

[10] Sjauw KD, Engström AE, Vis MM (2009). A systematic review and meta-analysis of intra-aortic balloon pump therapy in ST-elevation myocardial infarction: should we change the guidelines?. Eur Heart J.

[11] Thiele H, Zeymer U, Neumann FJ (2012). Intraaortic balloon support for myocardial infarction with cardiogenic shock. N Engl J Med.

[12] Wong CK, Gao W, Stewart RA, van Pelt N, French JK, Aylward PE, White HD (2006). Risk stratification of patients with acute anterior myocardial infarction and right bundle-branch block: importance of QRS duration and early ST-segment resolution after fibrinolytic therapy. Circulation.

[13] Rumana N, Kita Y, Turin TC (2008). Trend of increase in the incidence of acute myocardial infarction in a Japanese population: Takashima AMI Registry, 1990-2001. Am J Epidemiol.

[14] Hata J, Ninomiya T, Hirakawa Y (2013). Secular trends in cardiovascular disease and its risk factors in Japanese: half-century data from the Hisayama Study (1961-2009). Circulation.

[15] Ettehad D, Emdin CA, Kiran A (2016). Blood pressure lowering for prevention of cardiovascular disease and death: a systematic review and meta-analysis. Lancet.

[16] Colhoun HM, Betteridge DJ, Durrington PN (2004). Primary prevention of cardiovascular disease with atorvastatin in type 2 diabetes in the Collaborative Atorvastatin Diabetes Study (CARDS): multicentre randomised placebo-controlled trial. Lancet.

[17] Cholesterol Treatment Trialists' (CTT) Collaborators; Kearney PM, Blackwell L, Collins R (2008). Efficacy of cholesterol-lowering therapy in 18,686 people with diabetes in 14 randomised trials of statins: a meta-analysis. Lancet.

[18] (1998). Effect of intensive blood-glucose control with metformin on complications in overweight patients with type 2 diabetes (UKPDS 34). UK Prospective Diabetes Study (UKPDS) Group. Lancet.

[19] Zinman B, Wanner C, Lachin JM (2015). Empagliflozin, cardiovascular outcomes, and mortality in type 2 diabetes. N Engl J Med.

[20] Emdin CA, Rahimi K, Neal B, Callender T, Perkovic V, Patel A (2015). Blood pressure lowering in type 2 diabetes: a systematic review and meta-analysis. JAMA.

